# Effectiveness of Goreisan in Ménière’s Disease

**DOI:** 10.7759/cureus.88643

**Published:** 2025-07-24

**Authors:** Yuki Koda

**Affiliations:** 1 Otolaryngology-Head and Neck Surgery, Osaka Metropolitan University, Osaka, JPN

**Keywords:** dizziness handicap inventory, goreisan, kampo medicine, ménière’s disease, vertigo frequency

## Abstract

Objective: This study evaluates the effectiveness of Goreisan, a traditional Kampo medicine, in treating Ménière’s disease (MD), focusing on vertigo frequency, dizziness severity, and hearing function.

Methods: A retrospective analysis was conducted on 164 patients diagnosed with definite MD between April 2017 and March 2022. Among them, 52 patients received Goreisan treatment, while 31 patients did not receive any diuretic therapy. Vertigo frequency, Dizziness Handicap Inventory (DHI) scores, and hearing function were assessed over a 12-month period.

Results: Patients in the Goreisan group showed a significant reduction in vertigo frequency at two, six, and 12 months, whereas the no-medication group showed significant improvement only at two months. DHI scores improved in both groups, suggesting that betahistine and lifestyle modifications may contribute to symptom relief. No significant changes in hearing function were observed in the Goreisan group.

Conclusion: Goreisan may be an effective and safe treatment option for reducing vertigo frequency in MD patients. However, it does not appear to improve hearing function. Further placebo-controlled studies are needed to confirm its efficacy.

## Introduction

Ménière’s disease (MD) is characterized by recurrent vertigo attacks accompanied by auditory symptoms such as hearing loss, tinnitus, and aural fullness. In Japan, conservative treatment during the intermittent period primarily consists of lifestyle guidance and pharmacotherapy. Recommended pharmacotherapies include osmotic diuretics, antivertigo drugs, anxiolytics, vitamin B12, and Kampo medicine. Among osmotic diuretics, isosorbide is frequently used. Several reports suggest the use of Goreisan, a traditional Kampo medicine with diuretic properties, for MD treatment [1].

Goreisan is composed of five essential herbal ingredients with water-regulating effects: *Poria*, *Atractylodes lancea*, *Polyporus*, *Alismatis*, and cinnamon bark. It is traditionally used to treat thirst, edema, nephrosis, hangovers, acute gastroenteritis, diarrhea, nausea, vomiting, gastric retention, headaches, uremia, and diabetes, and it is widely applied in internal medicine. Unlike Western diuretics, Goreisan increases urine output without significantly altering plasma electrolyte concentrations. Moreover, its diuretic effect is observed only in edematous states, while it does not promote excessive diuresis in dehydrated conditions [2-6]. This makes Goreisan a clinically convenient Kampo medicine.

Although there are some reports on its use in MD treatment, comprehensive studies are lacking. In this study, we conducted a retrospective analysis. This study aimed to evaluate two primary aspects. First, we compared Ménière’s disease (MD) patients who were treated with Goreisan to those who did not receive any diuretic therapy, focusing on changes in vertigo frequency and vertigo severity. The severity of vertigo was assessed using the Dizziness Handicap Inventory (DHI), a validated questionnaire that quantifies the impact of dizziness on daily life. Second, we investigated the longitudinal changes in hearing function among patients who received Goreisan, assessing whether hearing levels were preserved or altered over time.

## Materials and methods

Between April 2017 and March 2022, a total of 164 patients diagnosed with definite Ménière’s disease (MD) at our department’s vertigo outpatient clinic were enrolled. Among them, 52 patients received Goreisan treatment (Goreisan group), while 31 patients did not receive any diuretic treatment (no-medication group). Patients in the Goreisan group who were also receiving osmotic or other diuretics were excluded from the study. The Goreisan group consisted of 12 men and 40 women, with a mean age of 56±14 years. The affected ear was the right in 18 cases, the left in 28 cases, and bilateral in six cases. In the no-medication group, there were 13 men and 18 women, with a mean age of 53±14 years. The affected ear was the right in 10 cases and the left in 21 cases. All patients were administered betahistine as an antivertigo medication.

The diagnosis of Ménière’s disease (MD) was made according to the 2011 Japanese Society for Equilibrium Research clinical guidelines for cases diagnosed until May 2020 and the 2020 guidelines for cases diagnosed thereafter [1].

Goreisan was administered as a powdered extract at a total daily dose of 7.5 g, divided into three doses taken before each meal. Due to the retrospective nature of this study, adherence was assessed based on medical records and patient interviews during follow-up visits, without direct measures such as pill counts.

The evaluation included three main components. First, the mean monthly vertigo frequency was assessed at four time points: baseline (zero months), two months (mean frequency from zero to two months), six months (mean frequency from two to six months), and 12 months (mean frequency from six to 12 months). Second, dizziness-related quality of life was evaluated using the Dizziness Handicap Inventory (DHI) score. Third, hearing function was evaluated in the Goreisan group only. A total of 52 patients (58 ears, including bilateral cases) were assessed. Hearing thresholds were measured at 125 Hz, 250 Hz, and 500 Hz at zero, two, six, and 12 months.

Patients with incomplete outcome data at any follow-up time point were excluded from analyses pertaining to that specific time point. No imputation methods were applied to missing data.

The baseline demographic characteristics, including sex, affected side, age, Dizziness Handicap Inventory (DHI) score, mean monthly vertigo frequency, and hearing levels, are summarized in Table 1.

**Table 1:** Patient Background Comparison between the Goreisan treatment group and the no-medication group. Goreisan treatment group, 52 cases; no-medication group, 31 cases. Statistical analysis was performed using the Mann-Whitney U test (ns: non-significant) DHI: Dizziness Handicap Inventory

	Goreisan Treatment Group (n=52)	No-Medication Group (n＝31)	p
Sex ratio (male/female)	12:40	13:18	-
Affected side (right/left/bilateral)	18:28:06	10:21	-
Age (mean±SD) years	56±14	53±14	ns
DHI score (initial consultation)	46±27	35±24	ns
Mean monthly vertigo frequency (initial consultation)	5	1	0.018
Hearing levels (125 Hz)	41±19	24±20	ns
Hearing levels (250 Hz)	43±18	29±21	ns
Hearing levels (500 Hz)	39±19	24±11	ns
Hearing levels (1000 Hz)	33±20	22±21	ns
Hearing levels (2000 Hz)	30±19	20±18	ns
Hearing levels (4000 Hz)	36±22	21±9	ns
Hearing levels (8000 Hz)	48±27	27±12	ns

All statistical analyses were performed using IBM SPSS Statistics Base 25 (IBM Corp., Armonk, NY). Both t-tests and Mann-Whitney U tests were applied as appropriate. A two-sided p-value of less than 0.05 was considered statistically significant.

This study was approved by the institutional ethics committee of Osaka Metropolitan University (approval number: 2020-082).

## Results

**Figure 1:**
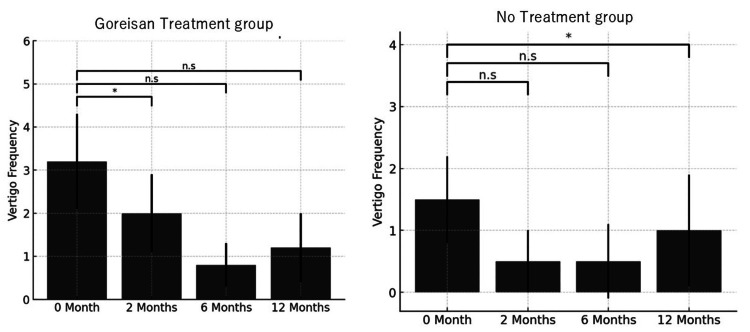
Comparison of Mean Monthly Vertigo Frequency at Zero, Two, Six, and 12 Months: Goreisan Treatment Group Versus No-Treatment Group In the Goreisan group, a significant reduction was observed at two months, six months, and 12 months compared to zero months. In the no-treatment group, a significant reduction was observed at two months, but no significant difference was found at six months and 12 months. Statistical analysis was performed using the Mann-Whitney U test (ns: non-significant; *p<0.05)

The mean monthly frequency of vertigo episodes demonstrated notable differences between the Goreisan treatment group and the no-treatment group over the 12-month observation period, as shown in Figure 1. In the Goreisan group, the frequency of vertigo episodes significantly decreased at two months (p<0.05), six months (p<0.05), and 12 months (p<0.05) compared to baseline (zero months). This pattern indicates a sustained and progressive therapeutic benefit of Goreisan in controlling vertigo symptoms. The reduction was particularly marked at six and 12 months, suggesting that the effect of Goreisan may accumulate over time or that continued use contributes to the better long-term management of Ménière’s disease symptoms. It should be noted that the Goreisan group had a significantly higher baseline vertigo frequency than the no-treatment group, which may partly influence the magnitude of the observed reductions. This potential baseline imbalance is discussed further below.

In contrast, patients in the no-treatment group showed a significant reduction in vertigo episodes only at two months (p<0.05), with the frequency returning closer to baseline levels at six months (p=0.069) and 12 months (p=0.244). This suggests a possible short-term natural fluctuation or placebo effect early in the course, without a lasting improvement in vertigo control. These findings imply that, while spontaneous remission may occur temporarily, sustained reduction in vertigo attacks may be more reliably achieved with Goreisan therapy.

**Figure 2:**
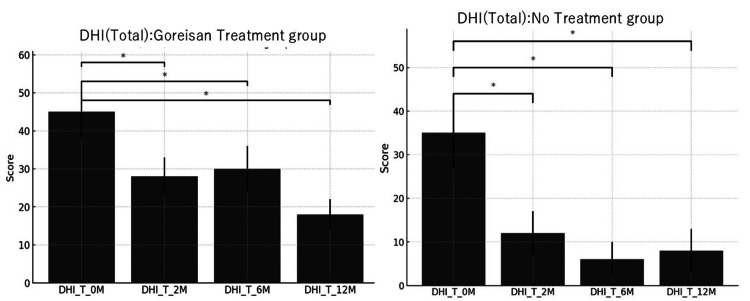
Longitudinal Comparison of Mean Dizziness Handicap Inventory Scores at Zero, Two, Six, and 12 Months: Goreisan Treatment Group Versus No-Treatment Group The Goreisan group showed a significant reduction in DHI scores at two months, six months, and 12 months compared to zero months. The no-medication group showed a significant reduction in DHI scores at two months, six months, and 12 months compared to zero months. Statistical analysis was performed using the Mann-Whitney U test (*p<0.05) DHI: Dizziness Handicap Inventory

The impact of treatment on dizziness-related disability was assessed using the Dizziness Handicap Inventory (DHI), and the results are summarized in Figure 2. Both groups demonstrated significant improvements in DHI scores at two, six, and 12 months compared to baseline (all p<0.05), indicating a general decrease in the subjective perception of dizziness and its effect on daily functioning. However, the degree and persistence of improvement varied between groups. The Goreisan group showed a consistent downward trend in DHI scores, suggesting a potential therapeutic effect beyond symptom frequency, possibly linked to better functional adaptation or psychological benefit from symptom relief. The no-treatment group also improved, which may reflect spontaneous resolution, psychological adaptation, or response to follow-up attention. While both groups benefited, the mechanisms behind improvement may differ, therapeutic in the Goreisan group and potentially compensatory or psychological in the control group.

**Figure 3:**
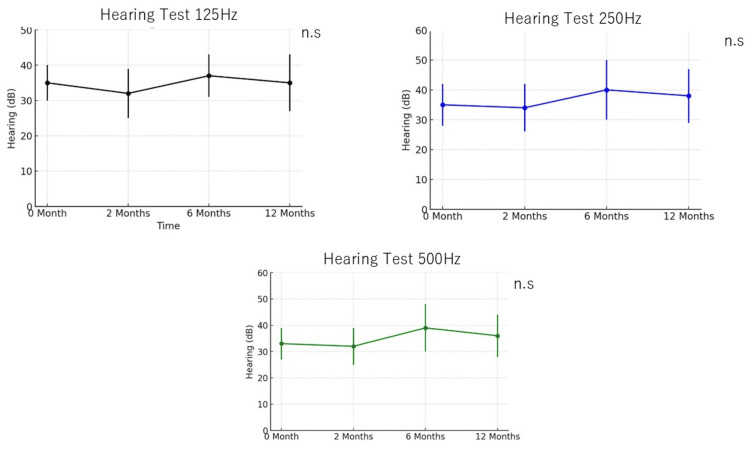
Longitudinal Comparison of Mean Hearing Thresholds at 125, 250, and 500 Hz at Zero, Two, Six, and 12 Months in Patients Treated with Goreisan Hearing levels were measured at 125 Hz, 250 Hz, and 500 Hz at zero months, two months, six months, and 12 months. No significant changes were observed. Statistical analysis was performed using the Mann-Whitney U test (ns: non-significant)

Low-frequency hearing thresholds at 125 Hz, 250 Hz, and 500 Hz were also examined exclusively in the Goreisan treatment group to evaluate any otological benefits of the intervention, as depicted in Figure 3. Across the two-, six-, and 12-month follow-up intervals, no significant changes were observed at any of the evaluated frequencies compared to baseline (p>0.05 at all points). This indicates that while Goreisan may be effective in controlling vertigo and improving the quality of life as reflected by DHI, it does not appear to significantly impact low-frequency auditory function in the short term to the midterm. The stability of hearing thresholds suggests that Goreisan neither improves nor worsens cochlear function during the treatment period, which may be relevant for assessing the overall safety profile of the intervention.

## Discussion

Ménière’s disease (MD) is characterized by endolymphatic hydrops. The Japanese MD treatment guidelines recommend a stepwise approach including lifestyle modifications, psychological interventions, and pharmacotherapy (osmotic diuretics, antivertigo drugs, anxiolytics, vitamin B12, and Kampo medicine). European guidelines suggest that a low-sodium diet and increased water intake may help maintain inner ear homeostasis by inhibiting vasopressin release [7]. The AAO-HNS guidelines recommend caffeine restriction in MD patients due to possible sympathetic nervous system stimulation affecting endolymph volume [8].

Osmotic diuretics such as isosorbide are widely used in Japan, although high-quality evidence for their efficacy is limited, and placebo-controlled trials are needed. Goreisan, unlike Western diuretics, selectively increases urine output in edematous states without altering electrolyte balance. This effect is believed to be mediated by aquaporins (AQPs), which regulate water transport. Previous studies suggest that Goreisan inhibits AQP3, AQP4, and AQP5. AQP1-5 are localized in the cochlea; AQP1 and AQP4 in the vestibular system; and AQP1-4 and AQP6 in the endolymphatic sac. These findings suggest that Goreisan may help reduce endolymphatic hydrops by modulating AQPs in the inner ear [9-13].

This study found that Goreisan significantly reduced vertigo frequency, while DHI scores improved in both groups. Improvement in the no-treatment group may be due to natural disease progression, betahistine use, and lifestyle changes. Importantly, the Goreisan group’s higher baseline (zero months) vertigo frequency might partly explain the magnitude of reduction observed; therefore, this potential baseline imbalance is acknowledged as a limitation. Hearing function did not improve significantly, consistent with prior reports showing that diuretics reduce vertigo but have minimal impact on hearing outcomes.

Furthermore, although we assessed hearing function only in the Goreisan group, which limits comparative analysis, this decision was made to focus on safety and feasibility within the retrospective design. We acknowledge that the absence of hearing data in the no-treatment group is a limitation that should be addressed in future studies.

While Goreisan appears to be a viable option for MD treatment, its effect on hearing loss remains unclear. Prospective placebo-controlled studies with adequate adjustment for confounders such as betahistine dosage, lifestyle adherence, and comorbidities are needed to confirm its efficacy and safety profile.

## Conclusions

Goreisan significantly reduced vertigo frequency in MD patients. DHI scores improved in both the Goreisan and no-treatment groups, indicating the role of betahistine and lifestyle modifications. No significant improvement in hearing function was observed. Goreisan’s favorable safety profile suggests that it is a promising treatment option for MD-related vertigo; further controlled studies are warranted.
